# Increased interstitial fluid in periventricular and deep white matter hyperintensities in patients with suspected idiopathic normal pressure hydrocephalus

**DOI:** 10.1038/s41598-021-98054-0

**Published:** 2021-10-01

**Authors:** Alexander Rau, Marco Reisert, Elias Kellner, Jonas A. Hosp, Horst Urbach, Theo Demerath

**Affiliations:** 1 Department of Neuroradiology, Medical Center – University of Freiburg, Faculty of Medicine, University of Freiburg, Freiburg, Germany; 2 Medical Physics, Department of Radiology, Medical Center – University of Freiburg, Faculty of Medicine, University of Freiburg, Freiburg, Germany; 3 Department of Neurology, Medical Center – University of Freiburg, Faculty of Medicine, University of Freiburg, Freiburg, Germany

**Keywords:** Diseases of the nervous system, Neurodegeneration, White matter disease

## Abstract

Periventricular white matter changes are common in patients with idiopathic normal pressure hydrocephalus (iNPH) and considered to represent focally elevated interstitial fluid. We compared diffusion measures in periventricular hyperintensities in patients with imaging features of iNPH to patients without. The hypothesis is that periventricular hyperintensities in patients with presumed iNPH show higher water content than in patients without imaging features of iNPH. 21 patients with iNPH Radscale 7–12 (“high probability of iNPH”) and 10 patients with iNPH Radscale 2–4 (“low probability of iNPH”) were examined with a neurodegeneration imaging protocol including a diffusion microstructure imaging sequence. Periventricular hyperintensities and deep white matter hyperintensities were segmented and diffusion measures were compared. In patients with imaging features of iNPH, the free water content in periventricular hyperintensities was significantly higher compared to the control group (*p* = 0.005). This effect was also detectable in deep white matter hyperintensities (*p* = 0.024). Total brain volumes and total gray or white matter volumes did not differ between the groups. Periventricular cap free water fraction was highly discriminative regarding patients with presumed iNPH and controls with an ROC AUC of 0.933. Quantitative diffusion microstructure imaging shows elevated water content in periventricular hyperintensities in patients with imaging features of iNPH, which could be the imaging correlate for pathologic fluid accumulation and may be used as an imaging biomarker in the future.

## Introduction

Periventricular hyperintensities (PVHs) are T2-hyperintense white matter areas located around the lateral (mostly frontal and occipital) ventricles, commonly found in standard MRI studies in the older population with and without idiopathic normal pressure hydrocephalus (iNPH)^1^.

Pathologically, they represent areas of finely textured myelin associated with denudation of the ventricular ependymal lining rather than ischemic or gliotic changes^2^. The ependymal lining is regarded as a bidirectional barrier and transport system for cerebrospinal fluid (CSF) and fluid exchange. Age-related ependymal denudation might play a pathophysiological role in age-related impaired fluid transport, which may result in parenchymal periventricular fluid accumulation^2^. In patients with iNPH, chronic CSF circulation disturbance has been demonstrated, e.g. by intrathecal Gadolinium administration and delayed clearance of intrathecal Gadolinium in perivascular spaces. This disturbance is considered to lead to increased capping due to CSF accumulation in PVHs in patients with iNPH^3,4^. As both age-related capping and periventricular interstitial edema show PVH with an increased T2 signal, they can usually not be differentiated from one another in conventional MRI^1^. In contrast, deep white matter hyperintensities (DWMH) are always considered pathological and occur in both iNPH and small vessel disease. Histopathologically, DWMH are considered primarily ischemic because of their similarity to thromboembolic infarcts, although blood–brain barrier disruption with interstitial fluid accumulation is also discussed as a cause^5^.

In iNPH, DTI was used to evaluate changes in white matter tracts and found increased fractional anistropy (FA), reduced mean diffusivity and increased oriental coherence in the corticospinal tract^6,7^ or decreased FA in the corpus callosum and increased FA in the internal capsule^8^ as reviewed by Siasios and colleagues^9^. In a recent study using a quantitative multi-echo, gradient-echo water mapping sequence in a cohort of patients with neurovascular symptoms an increased water content in periventricular caps compared to DWMH was described^10^. Though to date, focused evaluation of iNPH-associated T2w hyperintense white matter changes based on current diffusion sequences is missing.

Novel multi-compartment diffusion microstructure imaging (DMI) techniques have been used to characterize white matter meso-/microstructure. They promise to bridge the gap between classic in vivo MR imaging and post-mortem microscopy methods^11^. DMI is based on advanced multi-shell diffusion protocols that allow distinguishing different anatomical compartments by their diffusion properties. The random motion of water within axons is purely one-dimensional. On the other hand, motion in the extra-cellular matrix is along all directions in space, but hindered by organelles and membranes (Fig. 1). This fact is used to decouple their contributions to the signal and allow the estimation of compartment specific volume fraction^12–14^. Next to the intra- and extra-axonal compartment there is typically a third compartment with non-negligible volume: ‘free’ water or CSF compartment, which includes water molecules moving freely at the distance of the diffusion length (in the range of tenth of micrometers). It has recently gained attention in the context of the glymphatic system^15–17^, but also as a confounder of classical DTI studies^18^. Typically, this compartment is specific to ventricles and perivascular spaces, which is of particular interest in this study. In this work we use the Bayesian approach of Reisert and colleagues^13^ to estimate DMI parameters, which is a robust generalization of neurite orientation dispersion and density imaging (NODDI). Compared to classical diffusion tensor imaging (DTI) indices, the DMI indices offer more specific and interpretable measures of tissue integrity.

**﻿Figure 1:**
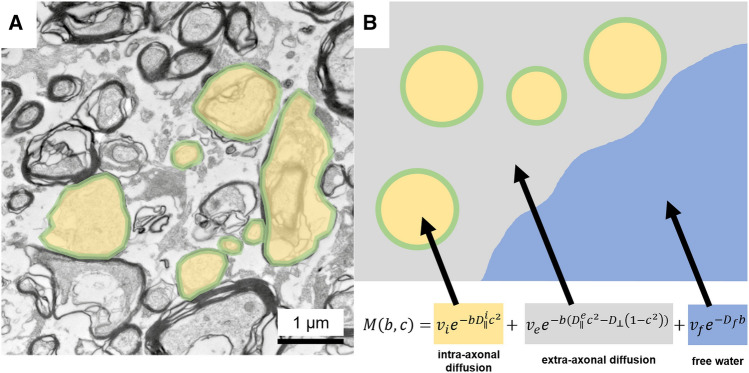
Diffusion Microstructure Imaging (DMI). White-matter electron microscopic (60 nm ultrathin sections, (**A**)) and schematic representation (**B**). In a three-compartment diffusion microstructure model, D and v describe the diffusivities and volume fractions of the corresponding compartments with the subscript **i** referring to the intraaxonal compartment (yellow), **e** to the extra-axonal compartment (grey) and **f** to the free water compartment (blue). Visualization of the extra-axonal and free water compartments are anatomically compromised by preparation artifacts in electron microscopy sections (**A**). Figure taken from: Demerath T, Donkels C, Reisert M, Heers M, Rau A, Schröter N, Schulze-Bonhage A, Reinacher P, Scheiwe C, Shah MJ, Beck J, Vlachos A, Haas CA, Urbach H. Gray-White Matter Blurring of the Temporal Pole Associated With Hippocampal Sclerosis: A Microstructural Study Involving 3 T MRI and Ultrastructural Histopathology. *Cereb Cortex*. 13:bhab320. doi: 10.1093/cercor/bhab320 (2021).

By comparing diffusion metrics in PVHs and DWMH we aim at a more detailed understanding of the underlying pathophysiology (i.e., primary (interstitial) fluid accumulation vs. primary gliotic changes) of PVHs in iNPH. Our hypothesis is (1) that PVHs in patients with imaging features of iNPH have different diffusion metrics which can be depicted with DMI, i.e. to show a higher water content than PVHs in patients without imaging features of iNPH. In addition (2), we suspect a centripetal gradient of these diffusion metric alterations originating from the ventricles.

## Materials and methods

### Patient cohort and MR imaging

**Figure 2:**
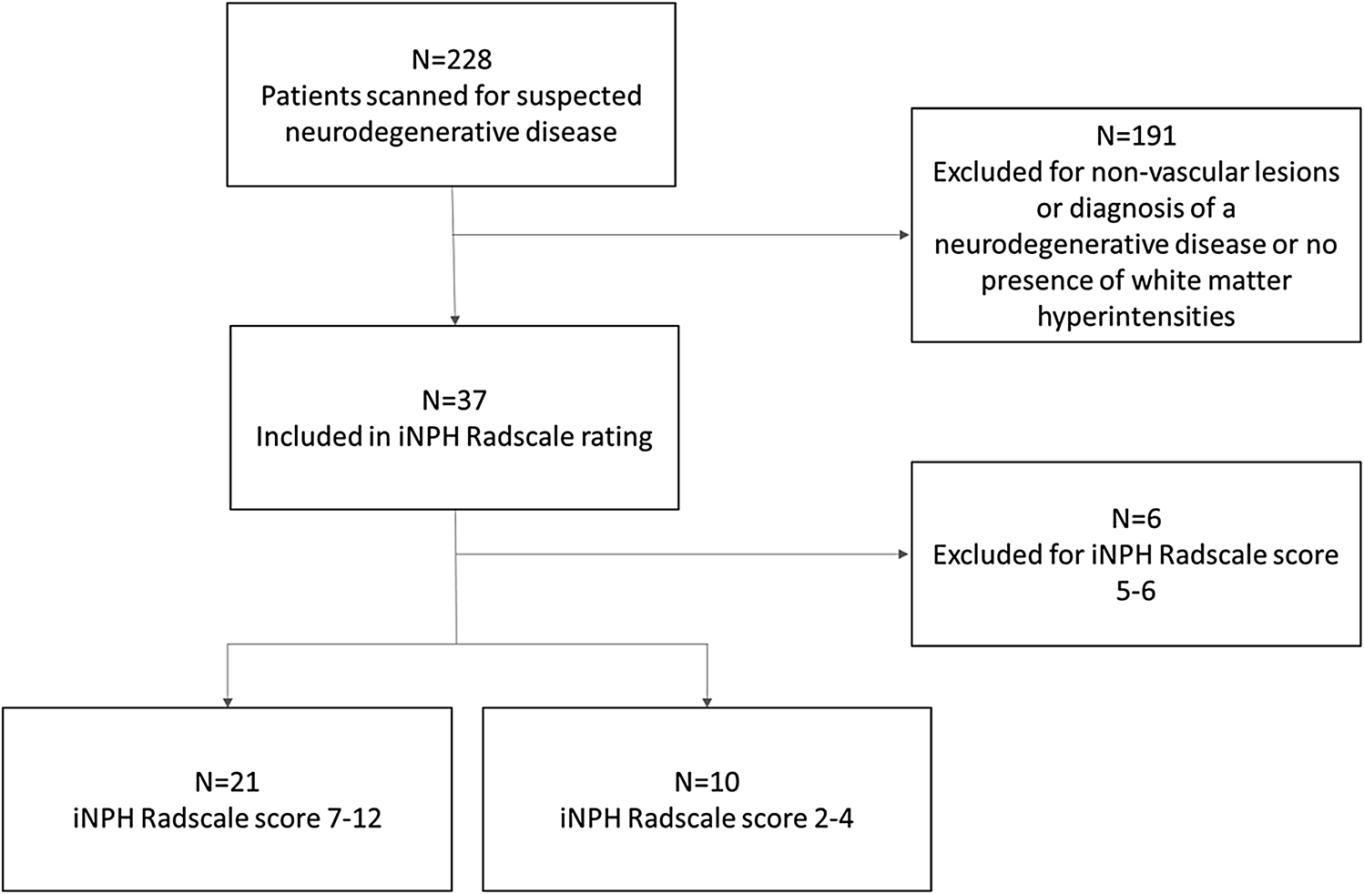
Summary of patient selection and group allocation.

Within a two-year period (2018–2019) 228 patients were referred due to a suspected neurodegenerative disease. Imaging was performed with a dedicated protocol for neurodegenerative diseases^19^ on a 3 Tesla scanner (MAGNETOM Prisma, Siemens Healthcare, Erlangen, Germany) using a 64 channel head and neck coil including a diffusion tensor/microstructure imaging (DTI/DMI) sequence (Supplementary Table 1). In this retrospective study, the prerequisite for inclusion in the further analysis was the presence of T2w-hyperintensities of the deep and also periventricular white matter. Patients with concomitant other non-vascular lesions (tumors, vascular malformations etc.) or other neurodegenerative disorders (e.g. Parkinson’s disease, atypical Parkinsonism, Alzheimer's disease, frontotemporal lobar degeneration (FTLD), cerebral amyloid angiopathy etc.) were excluded (see Fig. 2). MRI data were independently reviewed by two board certified neuroradiologists with > 4 and > 20 years of experience in clinical neuroimaging, blinded regarding diffusion metrics.

**Figure 3:**
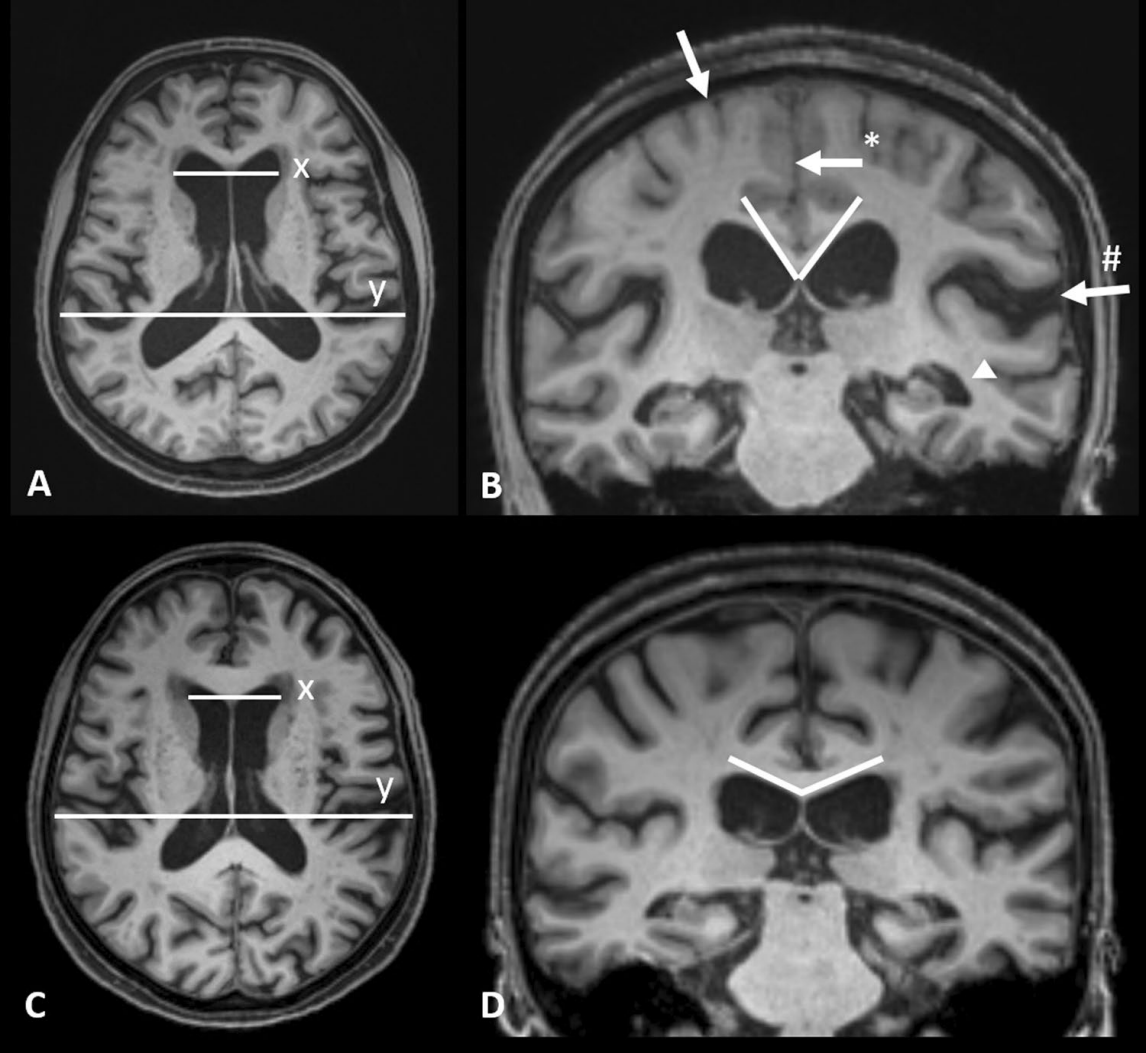
Axial and coronal T1 MPRAGE reformats in a 66 year old female patient with presumed iNPH (**A**, **B**) with iNPH Radscale 11: enlarged lateral ventricles (**A**, Evans index x/y = 0.31), widening of the Sylvian fissure (**B**, arrow#) and temporal horn of the lateral ventricles (arrowhead), narrowed parafalcine sulci (**B**, arrow*) and narrowed sulci along the vertex (**B**, arrow) and a callosal angle of 61° (white lines in **B**). 79 years old female control patient (**C**, **D**) with iNPH Radscale 4 for comparison: Evans index of 0.26 (**C**, x/y) and a callosal angle of 132° (**D**, white lines) in the absence of iNPH-typical features shown above.

Patients were classified based on the iNPH Radscale^20^ including typical iNPH imaging features such as: Increased Evans-Index (> 0.3)^21^, decreased callosal angle, measured at the level of the posterior commissure (< 90°)^22^, Disproportionately Enlarged Subarachnoid Space Hydrocephalus (DESH)^23^, narrowed sulci and a tight high convexity^24^, dilation of the temporal horns, focally enlarged sulci and periventricular white matter changes—an example is given in Fig. 3. Patients with iNPH Radscale scores of > 7 were assigned to the presumed iNPH-group. Patients scores of < 5 were assigned to the control group. Patients with intermediate scores of 5–6 were excluded from the analysis.

In addition, we evaluated this subjective scoring using an SVM-based approach^25^ using a prediction score of 0.42 [Range 0–1] as cut-off value, whereas > 0.42 corresponds to a high probability of iNPH and < 0.42 to a low probability of iNPH (in our study = control group).

The study is in accordance with the 1964 Helsinki Declaration and its later amendments and approved by the local ethical committee (Ethics Committee—Freiburg University Medical Center No. 400/20, approval 2020/08/13). Informed written consent was waived by the local ethics committee (Ethics Committee—Freiburg University Medical Center) due to the purely retrospective analysis. We hereby confirm that all methods were performed in accordance with the relevant guidelines and regulations.

### Diffusion microstructure imaging (DMI) and ROI based analysis

The DTI/DMI sequence parameters are given in Supplementary Table 1. All data processing was implemented within our in-house post-processing platform NORA (www.nora-imaging.org). Preprocessing of diffusion weighted images included a denoising step^26^ followed by correction of the Gibbs-ringing artifacts^27^ and final upsampling to isotropic resolution^13^.

**Figure 4:**
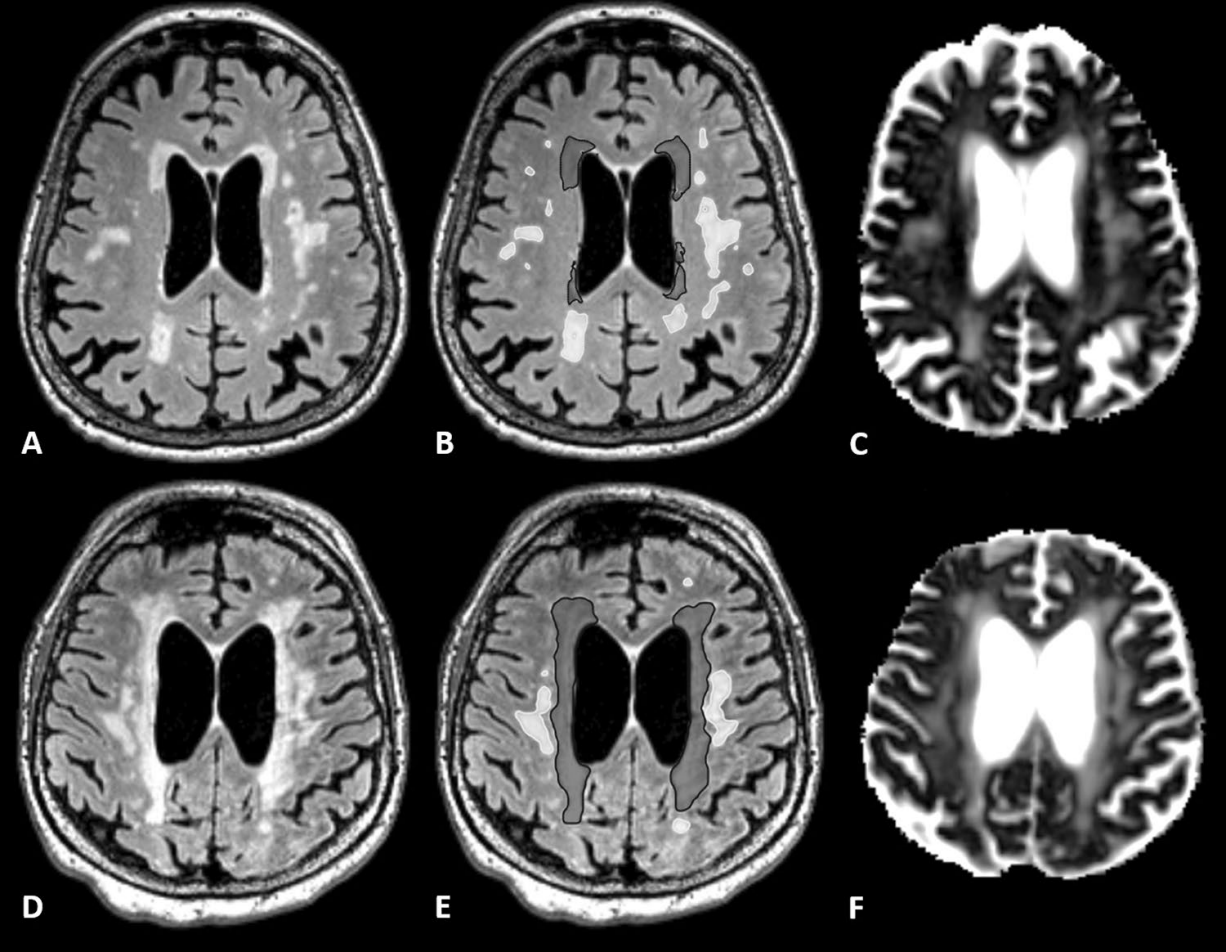
Axial FLAIR reformats in patients without (**A**) and with imaging features of iNPH (**D**) with corresponding superimposed segmentations (**B**, E) divided into periventricular hyperintensities (dark grey) and deep white matter lesions (light grey). Related parametric V-CSF-maps are shown in (**C**) and (**F**).

Microstructural diffusion metrics based on a three-compartment diffusion-model were estimated using a Bayesian approach^13^ to determine the intra-axonal volume fraction (V-intra), the extra-axonal intracellular volume fraction (V-extra) and the extracellular (CSF/ free water = V-CSF) volume fraction, respectively. T1w imaging datasets were automatically segmented into white matter, grey matter and cerebrospinal fluid (CSF) using CAT12 (http://www.neuro.uni-jena.de/cat/). White matter T2w-hyperintensities and periventricular hyperintensities (PVH) were manually segmented on 3D reformatted FLAIR images as depicted in Fig. 4. T2w hyperintensities adjacent to the ventricle surface were classified as PVHs and otherwise as DWMH according to the literature^28^. Furthermore, the lesion load was assessed using the Fazekas score^29^.

### Volumetry of total brain, CSF and ventricles.

The volumes of the created ROIs for PVH and DWMH were calculated. In addition, the total intracranial, total brain, gray and white matter volumes, total intracranial CSF and lateral ventricular volumes were calculated using an atlas-based approach^30^.

### Statistics

Continuous parametric variables are reported as mean and SD or median and ranges/interquartile ranges when appropriate. Normal distribution was tested with a Shapiro–Wilk test. The Mann–Whitney-U test and, when appropriate, 2-sided t-tests were performed to compare age, total brain, total gray and white matter, total intracranial CSF and lateral ventricle volumes, PVH and DWMH volumes between presumed iNPH and control groups. A One-way ANCOVA was conducted between PVH and DWMH comparing presumed iNPH and control groups, adjusting for age and lesion volume as covariates of no interest. A 2-sided Wilcoxon rank test was used to compare V-intra, V-extra and V-CSF within PVHs and DWMH within the groups. The Spearman correlation coefficient was used to relate PVH and DWMH V-CSF to lateral ventricular and lesional volumes. We plotted the ROC curves of PVH and DWMH V-CSF and the AUC and cut-off values were calculated. Values with an α-level of 0.05 were considered statistically significant. All statistical analyses were performed using R statistics version 3.6.3 (R Core Team, https://www.R-project.org). The Test ROC module was used to calculate ROC curves, which is built on the cutpointR module version 1.1.1 (https://CRAN.R-project.org/package=cutpointr).

### Ethics approval

All procedures performed in the studies involving human participants were in accordance with the ethical standards of the institutional and/or national research committee and with the 1964 Helsinki Declaration and its later amendments or comparable ethical standards. The Institutional Review Board approved this study (EK-Freiburg 400/20 approval 2020/08/13). Informed written consent was waived by the local ethics committee due to the purely retrospective analysis.

## Results

### Patient characteristics

We identified 37 patients with T2w-hyperintensities of the deep and also periventricular white matter. Of those, 21 patients fulfilled isolated imaging criteria for suspected iNPH (iNPH Radscale score 7–12; mean age 75, SD 6.4 years, 9 female) and 10 patients fulfilled imaging criteria for low iNPH Radscale scores (iNPH Radscale score 2–4; mean age 75, SD 10.2 years, 8 female). 6 patients with intermediate iNPH Radscale scores of 5–6 were excluded. Patients and imaging characteristics are given in Table 1. Since the Evans-index was a selection criterion, the lateral ventricle volume was significantly elevated in the group of presumed iNPH (*p* < 0.001). There was no significant difference in terms of age, total brain volume, volume of grey or white matter between the groups. There also was no significant difference regarding PVH (*p* = 0.142) and DWMH volumes (*p* = 0.990) between groups with imaging features of iNPH and controls, even though periventricular white matter changes are included in the selection criteria of the iNPH Radscale. The distribution of lesion load assessed by Fazekas score was similar between groups (Table 1), as about ¾ of patients within both groups presented with higher Fazekas scores (II-III).

**Table 1:** Patient and imaging characteristics.

	iNPH Radscale 7–12 (probable iNPH group)	iNPH Radscale 2–4 (control group)	*p*-value
n	21	10	
Gender (m/f)	12/9	2/8	
Age (years) (SD)	75 (6.4)	75 (10.2)	*p* = 0.982
CSF-volume (ml) (IQR)	572 (146.0)	474 (74.0)	*p* = 0.016
Lateral ventricle volume (ml) (IQR)	75.8 (14.8)	38.2 (18.0)	*p* < 0.001
Total brain volume (ml) (IQR)	932 (100.9)	956 (182.1)	*p* = 0.401
PVH volume (ml) (IQR)	12.1 (10.7)	5.6 (7.1)	*p* = 0.1
DWMH volume (ml) (IQR)	3.1 (4.2)	3.9 (3.5)	*p* = 0.724
Fazekas score (I/II/III)	6/9/6	3/4/3	

CSF = cerebrospinal fluid, DMI = diffusion microstructure imaging, DWMH = deep white matter hyperintensities, iNPH = idiopathic normal pressure hydrocephalus, PVH = periventricular hyperintensities. Values are given in mean and SD or median and interquartile ranges (IQR).

Using SVM analysis with a prediction score of 0.42 as cut-off value, the diagnosis of iNPH pattern was confirmed in 19/21 patients whereas 2/21 patients were assigned to the control group according to the SVM analysis. No patient in the control group was assigned to the iNPH group.

### DMI analysis

**Figure 5:**
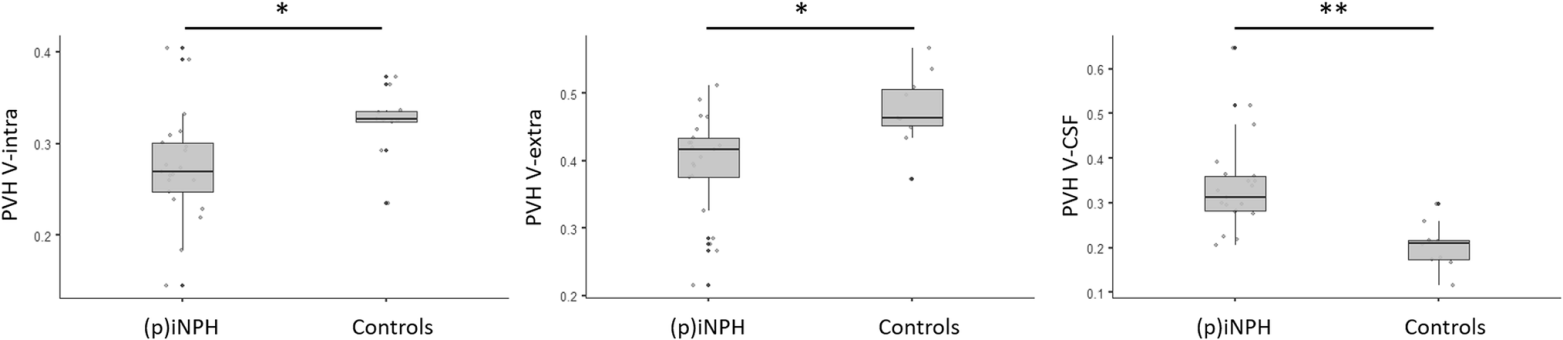
Periventricular cap (PVH) intraaxonal (**A**), extraaxonal (**B**) and CSF (**C**) volume fractions in patients with and without imaging features of idiopathic normal pressure hydrocephalus, defined by iNPH Radscale scores of > 6 ((presumed = p) iNPH; n = 21) and < 5 (Controls; n = 10). *p < 0.05, **p < 0.01.

DMI-parameters in PVH and DWMH were compared between groups with imaging features of iNPH and controls, adjusting for age and lesion volume. In PVHs, there was a significant reduction of V-intra in presumed iNPH compared to controls, F (1,1) = 5.06, *p* = 0.033, reduced V-extra, F (1,1) = 4.89, *p* = 0.036 and increased V-CSF, F (1,1) = 9.40, *p* = 0.005. In DWMH in presumed iNPH there was a significant reduction in V-intra, F (1,1) = 16.81, *p* < 0.001 with no significant difference in V-extra, F (1,1) = 0.16, *p* = 0.689 but a significant increase in V-CSF, F (1,1) = 5.68 *p* = 0.024. Group related metrics and ranges are presented in Table 2. Within-group comparisons between PVHs and DWMH showed no significant results. For details see Fig. 5.

**Table 2:** PVH- and DWMH-ROI derived diffusion metrics.

		iNPH Radscale 7–12 n = 21	iNPH Radscale 2–4 n = 10	*p*-value
PVH	V-intra (IQR)	0.270 (0.053)	0.326 (0.012)	*P* = 0.033
	V-extra (IQR)	0.417 (0.058)	0.463(0.053)	*P* = 0.036
	V-CSF (IQR)	0.314 (0.078)	0.210 (0.042)	*P* = 0.005
DWMH	V-intra (IQR)	0.253 (0.074)	0.311 (0.116)	*P* < 0.001
	V-extra (IQR)	0.415 (0.070)	0.422 (0.075)	*P* = 0.689
	V-CSF (IQR)	0.345 (0.109)	0.224 (0.124)	*P* = 0.024

CSF = cerebrospinal fluid, DWMH = deep white matter hyperintensities, iNPH = normal pressure hydrocephalus, PVH = periventricular hyperintensities, V-CSF = free water/CSF fraction, V-extra = extraaxonal volume fraction, V-intra = intraaxonal volume fraction. Values are given as median and interquartile ranges (IQR).

### Correlation and ROC analysis

Results of the Spearman correlation indicated a strong positive correlation between lateral ventricular volume and PVHs V-CSF (rs = 0.87, *p* < 0.001) and also between lateral ventricular volume and DWMH V-CSF (rs = 0.78, *p* < 0.001) within the group with imaging features of iNPH. Within the control group, there was a weak positive association between lateral ventricular volume and PVH V-CSF, without reaching significance (rs = 0.49, *p* = 0.154). No significant correlation between PVH or DWMH volume and PVH/DWMH V-CSF was observed in both groups.

**Figure 6:**
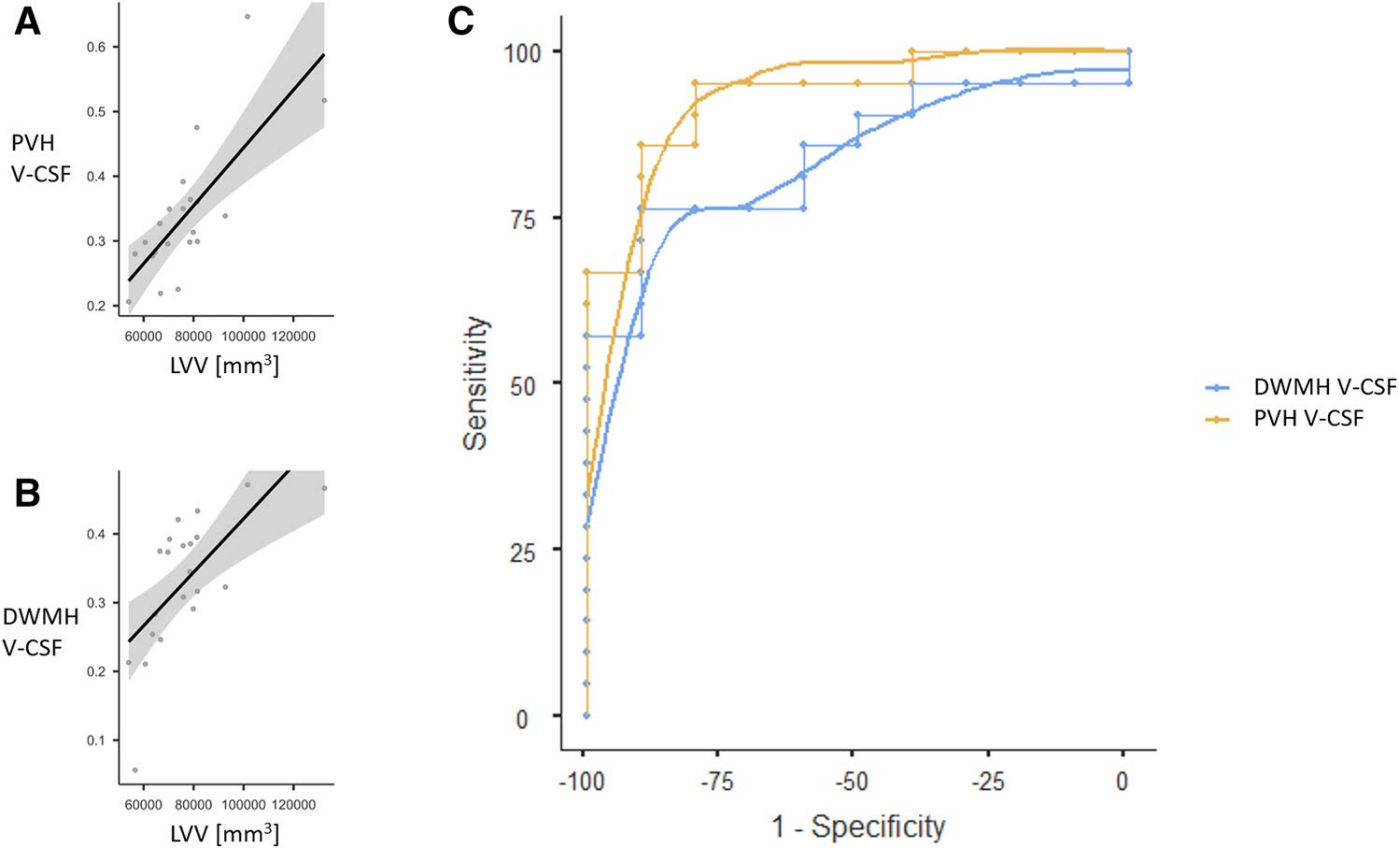
Scatterplots describing the associations between periventricular cap (PVH) V-CSF and lateral ventricular volume (LVV [mm^3^]; (**A**); deep white matter hyperintensity (DWMH) and LVV (**B**) in 21 patients with presumed iNPH. Receiver operating characteristic (ROC; **C**) of 21 patients with presumed iNPH and 10 controls showing high predictive value of both PVH V-CSF (AUC 0.933) and DWMH V-CSF (AUC 0.843) regarding iNPH defined by Radscale image criteria (Kockum et al.^40^).

Building on the systematic differences regarding free water content of PVH and DWMH between the group with imaging features iNPH and controls, we conducted a ROC analysis to determine a V-CSF threshold for optimal separation. A model equally weighted for sensitivity and specificity considerably supported the affiliation to the cohort with imaging features of iNPH for PVH V-CSF (sensitivity, 85.7%; specificity, 90.0%; PPV, 94.7%; NPV, 75.0%; AUC 0.933) when applying an estimated cutpoint of 0.277. DWMH V-CSF appeared only slightly less discriminative (sensitivity, 76.19%; specificity, 90.0%; PPV, 94.1%; NPV, 64.3%; AUC 0.843), applying an optimal cutpoint of 0.283. Correlation plots and ROC curves are presented in Fig. 6.

## Discussion

The pathogenesis of periventricular and deep white matter changes in iNPH is not fully understood. By analyzing diffusion metrics in periventricular hyperintensities in patients with imaging features of iNPH compared to those without we found a significant increase of the free water fraction (V-CSF). A similar, however less pronounced effect was observed in deep white matter hyperintensities (Fig. 5). Correspondingly, extra-axonal intracellular volume fraction (V-extra) and intra-axonal volume fraction (V-intra) were lowered. Within the cohort with imaging features of iNPH, there was also a strong correlation between PVH/DWMH free water fraction and lateral ventricular volume, which was not observed within the control group (Fig. 6A,B). We further investigated the potential predictive value of the free water fraction for high iNPH Radscale scores in an ROC analysis, which confirmed our previous results with an ROC AUC of 0.933 for PVH V-CSF and 0.843 for DWMH V-CSF (Fig. 6C).

A widely discussed pathomechanism of iNPH is impaired glymphatic function with reduction in CSF flow between the perivascular and interstitial spaces, resulting in decreased clearance of CSF from the subarachnoid space. Increased resistance to glymphatic flow may also cause CSF to follow the path of least resistance, among them, the retrograde transventricular route, resulting in an increased accumulation of CSF in the deep and periventricular white matter^4^. The increased free water fraction in PVHs in the group with imaging features of iNPH compared to controls in our study (Fig. 5) corroborates current results of previous studies examining the role of PVHs in patient populations with iNPH. It supports the hypothesis of increased (in particular periventricular) accumulation of free water in the brain parenchyma in iNPH patients^31^. The use of advanced diffusion imaging in this study allowed us to study this phenomenon non-invasively without the intrathecal application of Gadolinium and to elucidate the microstrucural changes in PVHs.

Beyond that, our data provide evidence that an increase in free fluid beyond the periventricular zone may be present in DWMH. This raises the question to what extent pathophysiological phenomena in iNPH exceed the immediate periventricular region. In line with our findings, it has been demonstrated that in iNPH reduced cerebral blood flow in the deep white matter extends beyond the periventricular areas^32^. Previous studies applying diffusion imaging techniques have also described related changes measuring FA and mean diffusivity within normal appearing white matter in patients with iNPH^6,33–35^. However, it is not yet clear whether the microstructural correlate of increased mean diffusivity in the pyramidal tract corresponds to increased water retention or microstructural white matter degeneration^6^. Another group has shown compartment shifts in iNPH using diffusion multicompartment imaging, revealing reduced axonal density within the CST in iNPH patients compared to controls^36^. In addition, one might ask whether a gradient of free water between periventricular and deep white matter could be present. However, lesion comparison (PVH vs. DWMH) within the group of patients with image features of an iNPH showed no significant difference. As we applied a pooled analysis of total volume in which we did not correct for individual lesion volume, voxel-based methods with intraindividual statistics and larger numbers of patients might be advantageous to test this hypothesis, especially since it is unknown how pronounced such a gradient could be.

Through intrathecal administration of gadobutrol it was shown that a tracer applied via the subarachnoid space propagated to deep brain tissue in a centripetal pattern and that clearance was delayed in the iNPH cohort^37^. This delayed clearance as well as the transependymal leakage might potentially result in increased levels of free water in DWMH, as we observed in our study. Whether centripetal CSF propagation or transependymal CSF leakage predominates in iNPH and whether there is a gradient between PVH and DWMH should be the subject of further studies.

The strong correlation of PVH V-CSF with ventricular width (Fig. 6A,B) suggests that there is a relationship between these phenomena in patients with imaging features of iNPH. Dilated ventricular spaces represent a hallmark of iNPH^22^ and thus are also part of diagnostic imaging criteria. However, dilated lateral ventricles (objectified, for example, with the Evan´s index) are a nonspecific phenomenon in older patients. Thus, the finding of ventriculomegaly alone is nonspecific and, for itself, nondiagnostic^38^.

In contrast, in our patient cohort, increased V-CSF in PVH and DWMH was strongly predictive for high iNPH Radscale scores. This indicates that a pathophysiological relationship may exist between dilated lateral ventricles and elevated levels of free fluid in the periventricular parenchyma in patients with imaging features of iNPH. The ROC analysis (Fig. 6C) revealed that a water volume fraction above 0.277 in periventricular lesions is strongly predictive for an iNPH imaging pattern, with an acceptable sensitivity and specificity.

The main limitation of our study is that due to the retrospective design the patient selection (iNPH) and the control group is based on imaging criteria alone, without considering clinical data (e.g., CSF pressure measurement), as recommended in current clinical diagnostic guidelines for iNPH^39^. Nevertheless, objectifiable criteria were applied using the iNPH Radscale, which showed low interrater variability in the evaluation based on CT and MRI and demonstrated a strong correlation with clinical features of iNPH in recent studies^40,41^.

Furthermore, based on the current literature, there is a transitional range in which individual imaging features of iNPH are present without clinically confirming the diagnosis of iNPH^42,43^. For this reason, patients with an intermediate iNPH Radscale score of 5–6 were excluded from the analysis. Therefore, the question of how far our findings correlate with clinical features of iNPH remains open and should be addressed in a larger, prospective cohort of iNPH patients. We believe that the diagnostic value of V-CSF for iNPH and shunt-responsiveness in particular should be further investigated. Another technical limitation is, that even though there is no difference in lesion volume between the groups, it cannot be ruled out that e.g., by inclusion of small hyperintensities partial volume effects might occur. This needs to be addressed by further (e.g., voxel-based) analyses in larger patient cohorts.

## Conclusion

In summary, diffusion microstructural imaging seems potentially valuable to further elucidate the role of white matter changes in iNPH. Using diffusion microstructure imaging, increased levels of free fluid in periventricular and deep white matter hyperintensities were observed in patients with imaging features of iNPH, positively correlating with increased lateral ventricular volumes. Whether this change is primarily due to white matter degeneration, malfunction of the cerebrospinal fluid circulation or both cannot be determined with certainty. Quantification of water content and intra-/extraaxonal volume fractions of periventricular T2 hyperintense white matter changes by diffusion microstructure imaging might be a useful additional tool for the differentiation of T2 hyperintensities in iNPH or other diseases.

## Supplementary Information

Supplementary Information.

## Data Availability

Not applicable.

## Abbreviations

CSF: Cerebrospinal fluid
DMI: Diffusion microstructure imaging
DWMH: Deep white matter hyperintensities
FA: Fractional anisotropy
iNPH: Idiopathic normal pressure hydrocephalus
PVH: Periventricular hyperintensities
SVM: Support Vector Machine
V-CSF: Free water/CSF fraction
V-extra: Extraaxonal volume fraction
V-intra: Intraaxonal volume fraction
