# Supplementary material for: Increased interstitial fluid in periventricular and deep white matter hyperintensities in patients with suspected idiopathic normal pressure hydrocephalus
Source: Sci Rep. 2021 Oct 1;11:19552. doi: 10.1038/s41598-021-98054-0 (PMC8486779; doi:10.1038/s41598-021-98054-0)
Supplement: Supplementary file 1 — Supplementary Information. [file 41598_2021_98054_MOESM1_ESM.docx]

**Supplementary Table 1**: Neurodegeneration-dedicated MRI protocol (3-Tesla MAGNETOM Prisma, Siemens Healthcare, Erlangen, Germany).

| **MRI sequence** | **No. of slices/ thickness (mm)** | **Voxel size (mm^3^)** | **TI/TR/TE/α**  **(ms/ms/ms/°)** | **acquisition time (min:sec)** |
| --- | --- | --- | --- | --- |
| **sag 3D MPRAGE** | 160/ 1 | 1x1x1 | 1100/2500/2.82/7 | 3:58 |
| **sag 3D FLAIR-SPACE** | 160/1 | 1x1x1 | 1800/5000/388/var | 6:52 |
| **ax 2D T2-TSE** | 42/3 | 0.4x0.4x3 | 5040/102/150 | 4:34 |
| **ax 2D T2-TSE** | 42/3 | 0.4x0.4x3 | 5040/102/150 | 4:34 |
| **ax 2D T2*** | 72/2 | 0.9x09x2 | -/27/20/17° | 2:39 |
| **DTI/DMI** | 42/3 | 1.5 x 1.5 x 3 | -/2800/88.5/90 | 6:22 |

MPRAGE Magnetization Prepared Rapid Gradient Echo, FLAIR SPACE Fluid-Attenuated Inversion Recovery - sampling perfection with application-optimized contrasts by using flip angle evolution, TSE Turbo Spin Echo, DMI Diffusion-Microstructure Imaging, SE Spin Echo, EPI Echo Planar Imaging, TI inversion time, TR repetition time, TE echo time, α flip angle
